# Effect of diabetic neuropathy severity classified by a fuzzy model in muscle dynamics during gait

**DOI:** 10.1186/1743-0003-11-11

**Published:** 2014-02-08

**Authors:** Ricky Watari, Cristina D Sartor, Andreja P Picon, Marco K Butugan, Cesar F Amorim, Neli RS Ortega, Isabel CN Sacco

**Affiliations:** 1 University of Sao Paulo; School of Medicine; Department of Physical Therapy, Speech and Occupational Therapy, Sao Paulo, SP, Brazil; 2 University of City of Sao Paulo (UNICID), Physical Therapy Master´s Program, Sao Paulo, SP, Brazil; 3 University of Sao Paulo, School of Medicine, Center of Fuzzy Systems in Health, Sao Paulo, SP, Brazil

**Keywords:** Diabetic neuropathies, Diabetes mellitus, Electromyography, Gait, Fuzzy logic

## Abstract

**Background:**

Electromyography (EMG) alterations during gait, supposedly caused by diabetic sensorimotor polyneuropathy, are subtle and still inconsistent, due to difficulties in defining homogeneous experimental groups with a clear definition of disease stages. Since evaluating these patients involve many uncertainties, the use of a fuzzy model could enable a better discrimination among different stages of diabetic polyneuropathy and lead to a clarification of when changes in muscle activation start occurring. The aim of this study was to investigate EMG patterns during gait in diabetic individuals with different stages of DSP severity, classified by a fuzzy system.

**Methods:**

147 subjects were divided into a control group (n = 30) and four diabetic groups: absent (n = 43), mild (n = 30), moderate (n = 16), and severe (n = 28) neuropathy, classified by a fuzzy model. The EMG activity of the vastus lateralis, tibialis anterior, and gastrocnemius medialis were measured during gait. Temporal and relative magnitude variables were compared among groups using ANOVA tests.

**Results:**

Muscle activity changes are present even before an established neural involvement, with delay in vastus lateralis peak and lower tibialis anterior relative magnitude. These alterations suggest an impaired ankle shock absorption mechanism, with compensation at the knee. This condition seems to be more pronounced in higher degrees of neuropathy, as there is an increased vastus lateralis activity in the mild and severe neuropathy groups. Tibialis anterior onset at terminal stance was anticipated in all diabetic groups; at higher degrees of neuropathy, the gastrocnemius medialis exhibited activity reduction and peak delay.

**Conclusion:**

EMG alterations in the vastus lateralis and tibialis anterior occur even in the absence of diabetic neuropathy and in mild neuropathic subjects, seemingly causing changes in the shock absorption mechanisms at the heel strike. These changes increase with the onset of neural impairments, and the gastrocnemius medialis starts presenting altered activity in the later stages of the disease (moderate and severe neuropathy). The degree of severity of diabetic neuropathy must be taken into account when analyzing diabetic patients’ biomechanical patterns of locomotion; we recommend the use of a fuzzy model for classification of disease stages.

## Background

Diabetic sensorimotor polyneuropathy (DSP) is believed to cause changes in muscle activity during gait. However, the literature has been inconsistent, with results that are not always reproducible, and which sometimes produce contradictions among studies, even when similar research methods are used. One potential explanation for these results is an imprecise and variable definition of diagnosis and classification criteria for DSP, leading to an over- or underestimation of the disease status [1]. Depending on the type of the assessment, authors may have allocated neuropathic individuals to groups considered free from this condition, and vice-versa.

Studies on lower limb EMG during gait in this population have resulted in insights regarding the generation and control of this motor task. Patients with DSP have shown delayed peak activity of the vastus lateralis and tibialis anterior at heel strike [2-4]; prolonged activity of the tibialis anterior associated with early onset of the soleus and gastrocnemius medialis during foot-flat phase [5], and delayed peak activity of the gastrocnemius lateralis and medialis at the propulsion phase [3,6]. Altered activity of the vastus lateralis and tibialis anterior have also been evident during stair negotiation [7], suggesting that these muscles play different roles when DSP is present. Although these findings are said to be connected to the presence of DSP, diabetic individuals without DSP also present differences in EMG patterns, such as prolonged activity of the vastus medialis at stance phase [8], and an anticipation of rectus femoris and gastrocnemius lateralis peak [9], which opposes the delayed activity found in patients with DSP [2,3,6].

Neurological signs and symptoms, and electrophysiological measurements are the most common methods for diagnosing DSP [10]. However, despite their higher reliability, nerve conduction studies are less accessible due to high cost, necessity of specialized professionals and equipment, and their occasionally invasive nature. Clinical assessment instruments provide simple and inexpensive options for the evaluation of DSP [11], but their results have shown to be variable when reproducibility and accuracy were analyzed [1,12,13], even when used as a composite score [13-15]. Furthermore, despite the possibility of combining different types of assessments, there are no objective criteria to interpret the association of those results, leaving the diagnosis to either a subjective decision by the health professional or a grading system that generates an outcome score by a simple sum.

In such a scenario, with unclear boundaries between sickness and health, the Theory of Fuzzy Sets can be a useful method for classifying these patients, as it takes into account the uncertainties of the clinical assessment of this illness, and it is capable of objectively measuring a subjective judgment. This tool could be useful for diagnosing and classifying DSP, as DSP evolves continuously from the onset of diabetes mellitus.

Duckstein et al. [16] have proposed a classification of this neurological disorder using fuzzy logic, but the input parameters they used were extracted from nerve conduction studies. Although it was an interesting approach, it relied on an invasive assessment method with accessibility limitations, and the sensitivity test was performed by comparing the fuzzy system’s output with the authors’ own classification criteria, leading to a possible bias. In another approach, Picon et al. [17] developed a fuzzy system, using clinical parameters as input variables, and they compared the output results with the opinions of health professionals who were DSP experts. However, even with good sensitivity and specificity, this system had a few drawbacks, such as overvaluation of disease symptoms on top of neurological signs, and the use of confounding factors, such as the last HbA1c level, a parameter that represents only a short period of the history of diabetes control.

The purpose of the present study was to investigate the lower limb EMG patterns of patients with different degrees of DSP severity, with each stage separated using a fuzzy model that uses simple clinical assessment methods as input variables. Our theses were that the use of fuzzy logic would enable a better distinction among the different stages of the disease and that muscle activation would be altered even before the onset of DSP, with increased effects in the more severe degrees of neuropathy.

## Methods

### Subjects

For this prospective study, 147 adult volunteers of both genders were divided into a control group of non-diabetic subjects (C; n = 30) and four diabetic groups, classified by means of a fuzzy system: non-neuropathic diabetic group (D; n = 43) and diabetic individuals with mild (MiN; n = 30), moderate (MoN, n = 16), and severe (SN; n = 28) neuropathy.

The inclusion criteria were: age under 65 years; absence of partial or total lower limb amputation or other neurological or orthopedic impairments due to stroke, cerebral palsy, poliomyelitis, rheumatoid arthritis, prosthesis, or moderate or severe osteoarthritis; major vascular complications (venous or arterial ulcers); severe retinopathy; severe nephropathy causing edema or requiring hemodialysis; absence of plantar ulcer at the time of evaluation; and ability to walk independently without pain or the use of an assistive device.

The research protocol was approved by the ethics committee of Faculdade de Medicina da Universidade de Sao Paulo (Protocol# 292/10), under the resolution 196/96 of the National Health Counsel, which is in accordance to the Declaration of Helsinki. All participants signed an informed consent form before entering the study.

All subjects underwent a clinical assessment, performed by a trained physical therapist, in which the following clinical parameters were evaluated: (a) vibratory perception with a 128 Hz tuning fork; (b) tactile sensitivity with a 10 g Semmes–Weinstein monofilament; and (c) typical neuropathy symptoms assessment, by means of a questionnaire based on the Michigan Neuropathy Screening Instrument [18]. These three groups of variables were used as linguistic inputs in a fuzzy model, based on Picon et al. [2012], to classify the patients into different disease severity stages. Only clinical assessment methods regularly used by health professionals, that are accessible and easily performable by any health professional were included in our evaluation.

Sociodemographic and clinical data of the experimental groups are described in Table 1. The groups differed in gender distribution, and all diabetic groups showed greater body mass index when compared to Group C. As expected, the duration of diabetes in Group D was shorter than in all the neuropathic groups; glycemia was higher in Group SN compared with D and MiN; and the neuropathy output score from the fuzzy model increased significantly as the degree of DSP increased.

**Table 1:** Sociodemographic variables and data related to diabetes mellitus for the experimental groups

	**Control (n = 30)**	**Diabetes no-neuropathy (n = 43)**	**Mild neuropathy (n = 30)**	**Moderate neuropathy (n = 16)**	**Severe neuropathy (n = 28)**	**p**
**Age (years)**	54.1 ± 7.5	56.7 ± 6.8	56.1 ± 6.3	58.4 ± 4.8	55.5 ± 6.8	0.346^1^
**Sex (% female)**	53.3	41.9	60.0	25.0	25.0	**0.027**^**3**^
**BMI (kg/m**^**2**^**)**	25.7 ± 3.9*	28.4 ± 3.9	28.5 ± 4.3	29.5 ± 4.6	28.6 ± 3.7	**0.009**^**1**^
**DM duration (years)**	-	8.1 ± 7.2*	12.1 ± 8.2	13.7 ± 5.9	14.4 ± 6.9	**0.003**^**2**^
**Glycemia (mg/dL)**	-	143.1 ± 56.6	156.5 ± 62.6	185.3 ± 98.1	197.9 ± 76.3^a^	**0,004**^**2**^
**Fuzzy neuropathy degree (score)**	-	0.9 ± 0.5*	2.9 ± 0.9*	5.9 ± 1.1*	8.9 ± 1.1*	**<0.001**^**2**^

^1^ANOVA; ^2^Kruskal-Wallis test; ^3^chi-square test *statistically different group; ^a^statistically different from diabetes and mild neuropathy; BMI – body mass index.

### Subject classification: Fuzzy system

**Figure 1:**
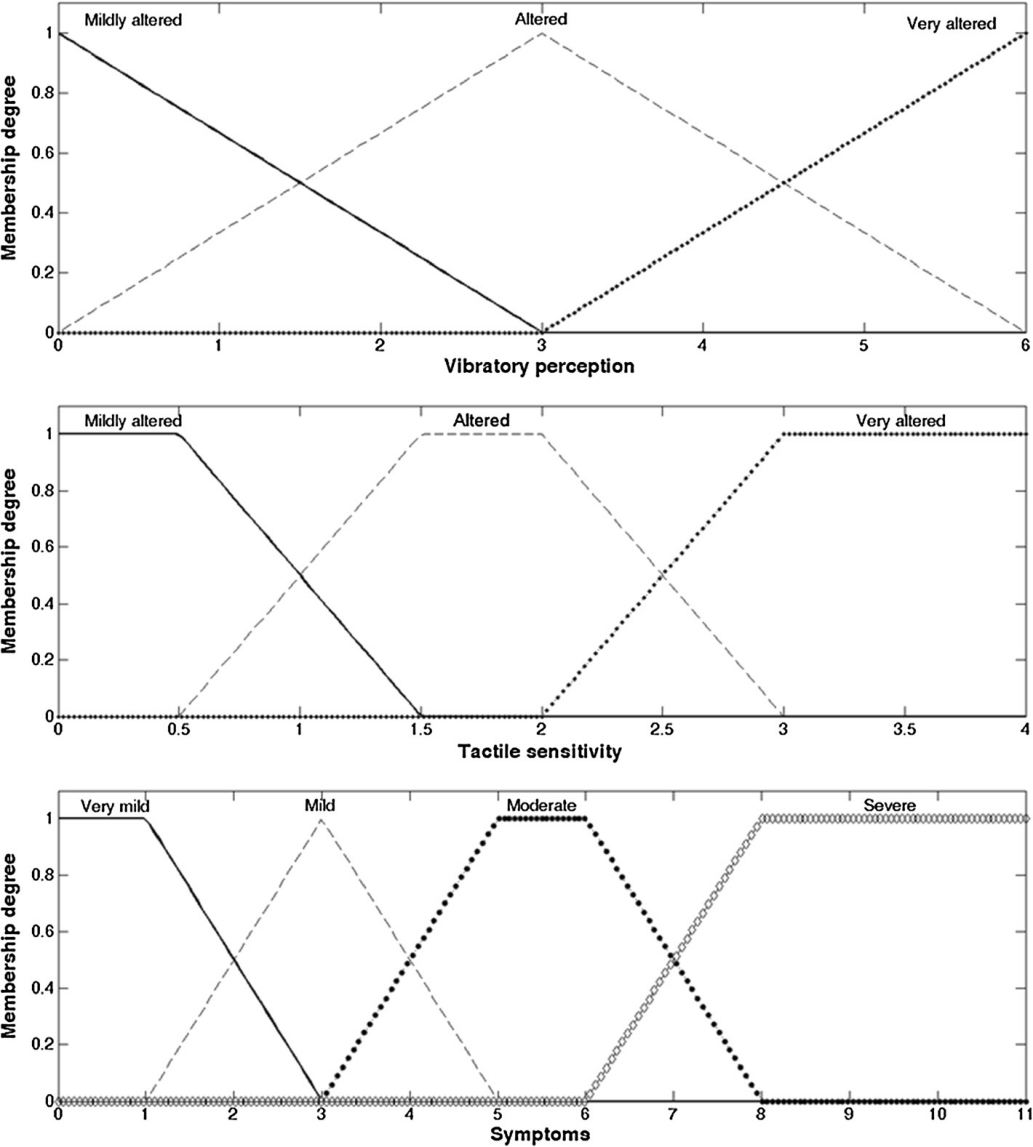
**Fuzzy sets of the system´s input variables.** Vibratory perception and tactile sensitivity were classified into: “*Mildly altered*”, “*Altered*” or “*Very altered*”. Symptoms were classified into: “*Very mild*”, “*Mild*”, “*Moderate*” or “*Severe*”. The graphical representation of each fuzzy set displays the membership degree that corresponds to a given value of the input variable.

**Figure 2:**
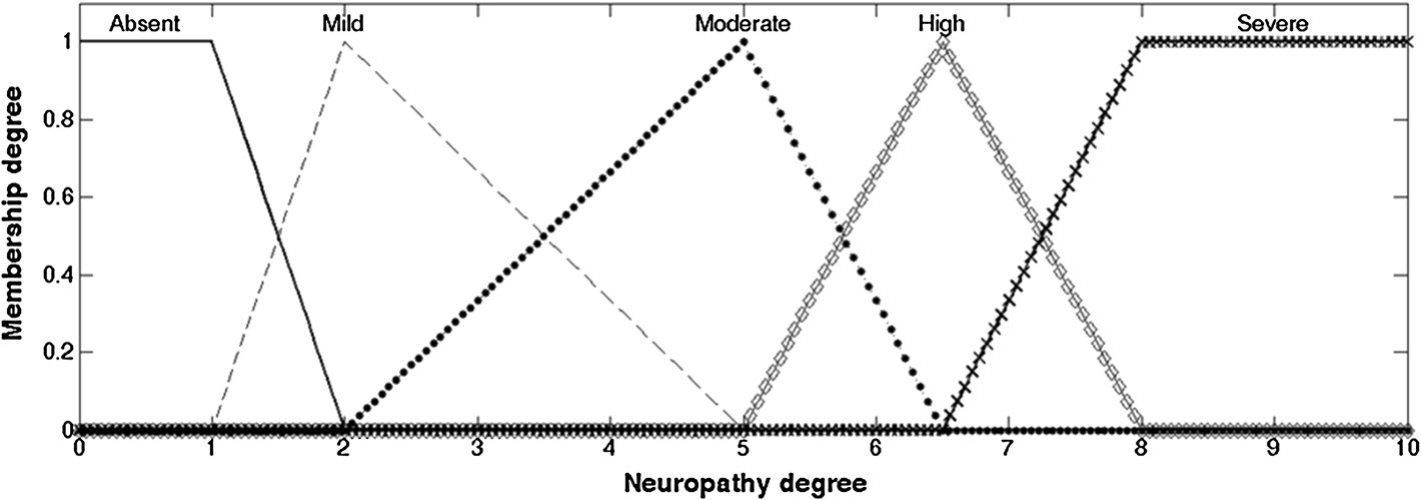
**Fuzzy output sets of the neuropathy degree.** Neuropathy degree was classified into: “*Absent*”, “*Mild*”, “*Moderate*”, “*High*” or “*Severe*”.

The fuzzy system classifies each input variables into fuzzy sets (fuzzification process) and performs a combinatory analysis of those variables by the Mamdani inference process [19], linking those combinations with fuzzy output sets. Then, by center of area defuzzification method, the resulting output sets are transformed into a numerical value. The model in this study used vibratory perception, tactile sensitivity, and symptoms assessment as the system´s inputs (Figure 1), and the combination among them determined the fuzzy output sets of the neuropathy degree (Figure 2) to which the subjects would be allocated, and the respective membership degree of each set. Finally, the defuzzification process produced the final “neuropathy degree score”. This value was sorted into the disease classes with the following division, with x being the score value: (i) x ≤ 2.5: absent neuropathy; (ii) 2.5 < x < 5.0: mild neuropathy; (iii) 5.0 ≤ x < 8.0: moderate neuropathy; (iv) x ≥ 8.0: severe neuropathy.

The fuzzy model presented a very strong correlation [20] with the experts’ opinion (Pearson’s coefficient r = 0.943) and a high accuracy level when classifying real patients that underwent the model’s analysis (ROC curve area = 0.91). More details of the system for classifying DSP may be found on the study of Picon et al. [17] in which the model used in this research was based on.

### Experimental procedures

The EMGs of the vastus lateralis (VL), tibialis anterior (TA), and gastrocnemius medialis (GM) muscles of one lower limb were measured during the stance phase of barefoot gait, as performed and described in our previous studies [6,7]. These muscles were chosen due to their role in gait progression (VL and GM), as well as knee and ankle impact attenuation (VL and TA) [21]. Furthermore, EMG activity of these muscles is often reported in gait studies [2,3,5,22], because there is less subcutaneous body fat-associated impedance, allowing a better comparison with the literature.

For data acquisition, an EMG system (model 800C; EMG System do Brasil, São José dos Campos, Brazil) with a signal amplification factor of 1000 was used. Disposable Ag/AgCl circular electrodes (10 mm diameter) were placed over each muscle with a center-to-center interelectrode distance of 20 mm, following the recommendations of Sacco et al. [23] for placement location, which is based on SENIAM. After shaving and cleaning the skin with alcohol, the electrodes were attached using Transpore 3M® adhesive tape and an elastic band, to avoid movement artifacts.

The subjects were instructed to walk at a self-selected cadence across a 10 m walkway with a force plate embedded in its center (model OR61000; AMTI, Watertown, MA). The electrode cables were held by a technician, who ensured that they were not overstretched or slapped against anything, while checking for any major gait deviation in ground reaction force during the data acquisition. EMG data were synchronized to the ground reaction force at a sampling rate of 2 kHz (A/D Board DT3002, 12 bits, AMTI), and five trials for each participant were collected. Data acquisition only started after a period of adaptation to the laboratory environment and equipment, when the subject demonstrated a regular gait pattern, without gross variation, confirmed by visual inspection of the vertical ground reaction force curve.

### Data processing

EMG activity was first processed to enable the extraction of linear envelopes. After removing the offset from the raw EMG, when necessary, the signal underwent a zero-lag fourth-order Butterworth filter with a band-pass width of 20–500 Hz, followed by full-wave rectification and low-pass filtering at 5 Hz. The linear envelope had its magnitude normalized by the EMG mean value and was time normalized by the stance phase period, determined by the ground reaction force data.

**Figure 3:**
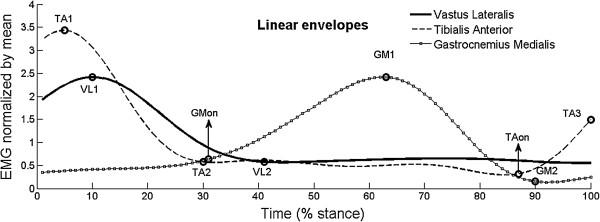
**Variables extracted from EMG linear envelopes.** Data extracted from the EMG linear envelopes for the calculation of the analyzed variables: peak and minimum activity of vastus lateralis (VL1 and VL2, respectively), tibialis anterior (TA1 and TA2) and gastrocnemius medialis (GM1 and GM2); activation magnitude of tibialis anterior at the end of stance phase (TA3), and onset of gastrocnemius medialis (GMon) and tibialis anterior (TAon).

The analyzed variables are shown in Figure 3: (i) relative magnitude of all three muscles, represented by the percentage of peak activity in relation to the minimum activity (VL1/VL2, TA1/TA2, and GM1/GM2); (ii) relative magnitude of TA at pushoff phase, represented by the percentage of the value of last peak activity in relation to minimum activity (TA3/TA2); (iii) time to peak occurrence (from 0 to 100% of stance phase) of all muscles (VL1, TA1, and GM1); and (iv) onset time [24] of GM (GMon) and of TA (TAon) in the terminal stance (last 25% of stance phase).

All data processing was performed with a custom-written MATLAB (version 7.8) function, and to ensure the validity of the computer-derived variables, each signal underwent visual inspection to identify possible movement artifacts or any other interference that could compromise the procedure.

### Statistical analysis

The EMG variables and sociodemographic and clinical data were tested for normal distribution with the Kolmogorov–Smirnov test. Inter-group comparisons of variables with non-normal distribution patterns were performed with the non-parametric Kruskal–Wallis test and the comparisons of normal distributed variables were performed by ANOVA tests followed by Neumann–Keuls post hoc (alpha error level of 5%). Gender distribution among groups was compared using a chi-square test. Statistics were performed with the Statistica software (v.9; StatSoft, Inc.).

## Results

**Figure 4:**
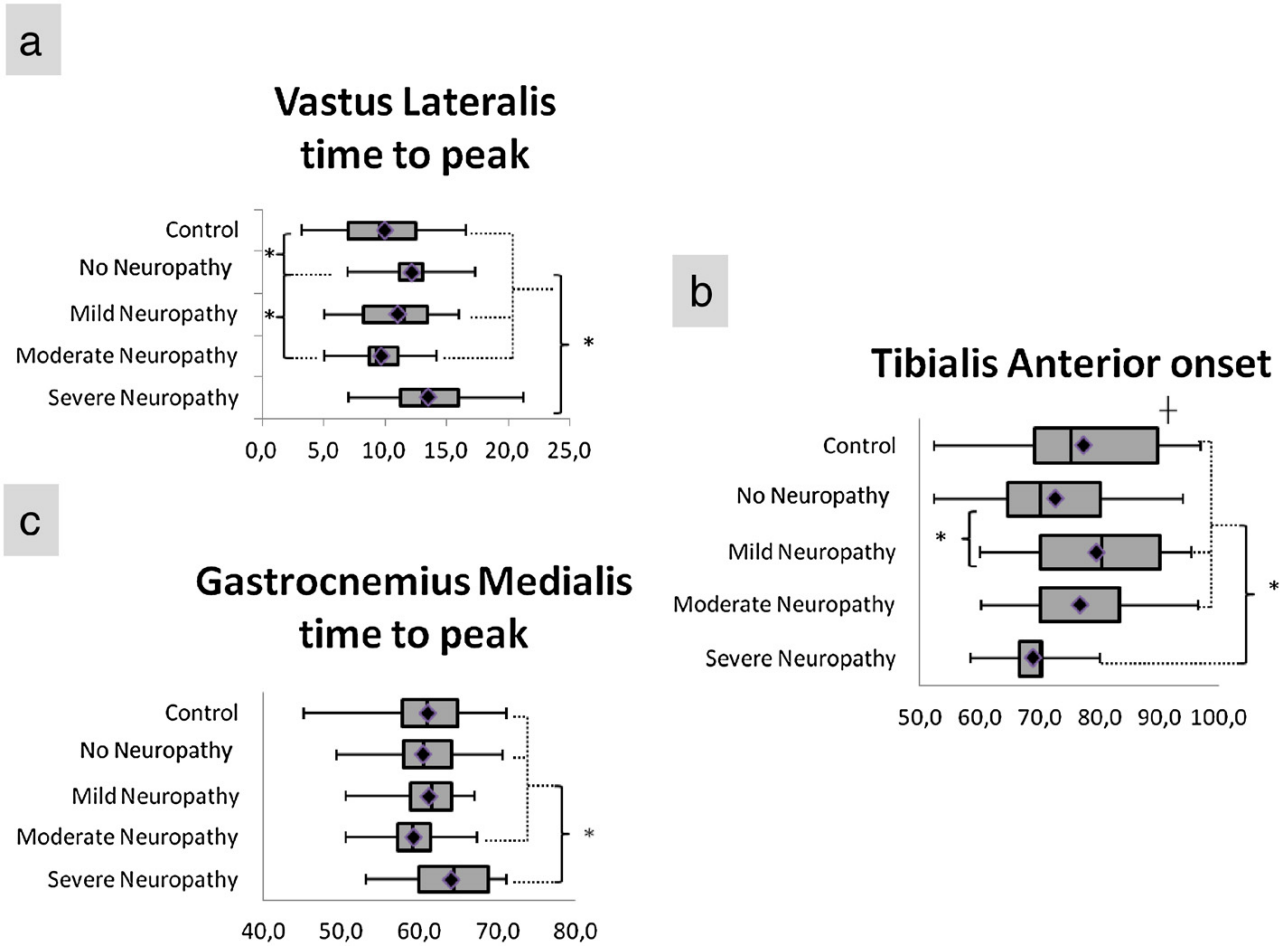
**Temporal variables with statistically significant differences.** Box plot with minimum-maximum range of **(a)** vastus lateralis time to peak occurrence; **(b)** tibialis anterior onset time; **(c)** gastrocnemius medialis time to peak occurrence. *Statistically significant difference between groups. ┼ Statistically different group.

**Figure 5:**
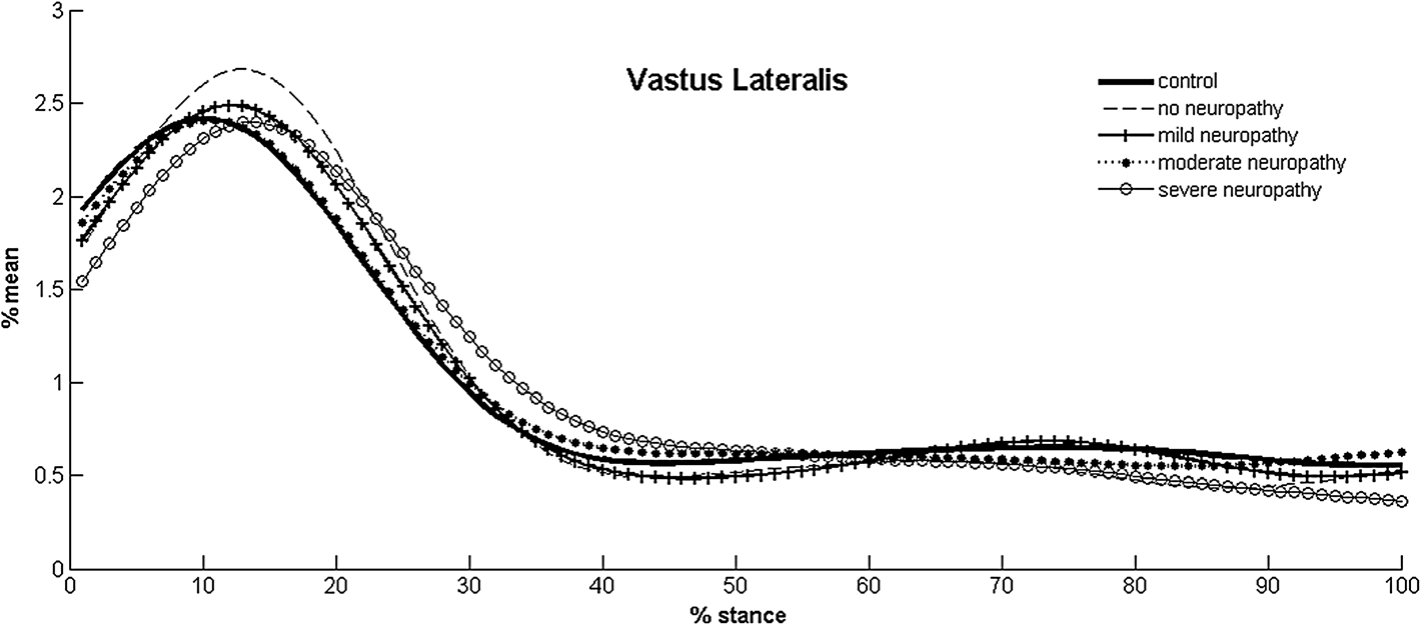
**EMG linear envelopes of vastus lateralis.** Mean linear envelopes of the vastus lateralis of all experimental groups, in which a qualitative analysis shows the delay in peak activity of no-neuropathy, mild and severe neuropathy groups.

All three muscles displayed changes in EMG activity at some point of the disease, but they did not occur with a defined pattern (Table 2, Figure 4). All the diabetic groups, except MoN, had a tendency to delay the peak activity of VL, although only Groups D and SN showed a significant difference compared to the other groups (Figure 5). Groups SN and MiN presented a higher relative magnitude of VL, while MoN had the lowest activity among diabetic groups.

**Table 2:** EMG temporal and relative magnitude variables (mag) for vastus lateralis (VL), tibialis anterior (TA) and gastrocnemius medialis (GM)

		**Control (n = 30)**	**Diabetes no-neuropathy (n = 43)**	**Mild neuropathy (n = 30)**	**Moderate neuropathy (n = 16)**	**Severe neuropathy (n = 28)**	**p**
**VL**	**Time to peak (% stance)**	9.7 ± 3.2*	12.1 ± 2.3*^§^	11.0 ± 3.3	9.7 ± 2.5^§^	13.5 ± 3.6^a^	**< 0.001**^1^
	**Mag (VL1/VL2)**	8.3 ± 4.0^b^	11.0 ± 6.5	13.6 ± 10.0*	6.8 ± 3.1^c^	17.1 ± 15.6*	**0.014**^2^
**TA**	**Time to peak (% stance)**	3.7 ± 2.0	4.2 ± 2.4	3.6 ± 2.1	2.2 ± 2.0	3.3 ± 2.6	0.055^1^
	**Mag (TA1/TA2)**	60.9 ± 55.5^d^	21.3 ± 12.8	24,7 ± 15.7	22.7 ± 20.6	33.4 ± 31.0	**0.043**^2^
	**Onset at push off (% stance)**	90.4 ± 6.1†	72.7 ± 10.8*	79.4 ± 12.5*	76.7 ± 10.5	68.8 ± 4.9^a^	**< 0.001**^2^
	**Mag at push off (TA3/TA2)**	6.0 ± 2.7*	8.1 ± 2.7*	7.1 ± 2.8	7.6 ± 2.0	7.1 ± 2.2	**0.014**^1^
**GM**	**Time to peak (% stance)**	60.0 ± 6.4	60.5 ± 5.1	61.3 ± 3.9	59.3 ± 4.3	64.0 ± 5.6^e^	**0.015**^**1**^
	**Mag (GM1/GM2)**	52.8 ± 48.8	33.7 ± 24.0	37.9 ± 24.5	19.2 ± 13.5^f^	16.0 ± 13.4^f^	**< 0.001**^**2**^
	**Onset (% stance)**	40.4 ± 5.4	37.3 ± 5.4	38.7 ± 8.5	34.8 ± 9.2	38.1 ± 6.1	0.089^1^

^1^ANOVA; ^2^Kruskal-Wallis test;

*, § statistically significant difference between groups; † statistically different group; ^a^statistically different from control, and mild and moderate neuropathy; ^b^statistically different from mild and severe neuropathy; ^c^statistically different from diabetes, and mild and severe neuropathy; ^d^statistically different from diabetes, and mild and moderate neuropathy; ^e^statistically different from control, diabetes and moderate neuropathy; ^f^statistically different from control, diabetes and mild neuropathy.

The TA relative magnitude was lower for all diabetic groups compared to Group C, although this comparison was not statistically significant for SN. The activation onset of TA at late stance occurred earlier in the diabetic groups, both with and without neuropathy, and it was more evident in Groups D and SN. Regarding the relative magnitude at the end of stance phase, Group C had the lowest activity overall, but only D presented a significantly higher value.

The GM relative magnitude had a tendency to be lower in the diabetic groups compared to Group C, with significant differences only for MoN and SN. Only Group SN showed a delay in GM peak activity, although the difference was not statistically significant when compared to MiN.

The delay in VL was approximately 26% for Groups D and SN, and these groups had a 28% anticipation of TA onset when compared to Group C, which was more pronounced than the 16% for Groups MiN and MoN (Figure 4). In Group SN, temporal change of GM peak was more subtle than in the other muscles, with an average of 6% delay (Figure 4).

## Discussion

The aim of this study was to investigate EMG patterns during gait in diabetic individuals with different stages of DSP severity, classified by a fuzzy system. The main findings indicate that diabetes mellitus and DSP are related to changes in muscle activity, although the alterations did not follow a distal to proximal order [25], nor did they progress in the same manner from mild to severe stages. In general, diabetic individuals display altered muscle activity even before the onset of DSP, suggesting an impaired shock absorption mechanism at the heel strike and flat-foot phases. In later disease stages, the muscles related to the propulsion phase are also affected.

Sacco et al. [2,4] and Akashi et al. [3] observed a delayed peak activation of VL in diabetic individuals with neuropathy, and in two studies, [3,4] this difference appeared only in patients with a history of plantar ulceration, an attribute that was presented by 84% of the subjects in the SN group, which displayed the same delay in the VL muscle. Therefore, neuropathy does seem to be related to this difference in temporal behavior of the VL, and once neural involvement starts, there is an additional change in the relative magnitude of VL, with an overall increase in the MiN and SN groups. Nonetheless, our findings show that this delayed pattern of VL is present even before the development of DSP, when only diabetes mellitus is present, indicating that these changes in VL might not be solely related to neural damage, but might also be influenced by other factors [26-28].

Diabetes mellitus itself is related to alterations in muscle cells due to hyperglycemia, insulin resistance, and accumulation of advanced glycation end-products. These effects lead to reduced nerve conduction velocity [26], loss of muscle mass, reduced muscle cross-sectional area, muscle protein degradation [27], and loss of muscle strength [28], which could be one of the underlying causes for most of our findings that occurred in subjects without neuropathy (D group).

The interesting VL results observed in the diabetic subjects can be based on the findings of Petrofsky et al. [29] and Ko et al. [30]. In the Petrofsky et al. [29] study on gait, diabetics without sensory or muscle strength deficits presented knee joint acceleration two times greater at the heel strike than healthy individuals. Furthermore, these diabetic individuals have shown higher absorptive mechanical work expenditure at the knee joint and a delay on the instant of the first knee flexion peak in stance phase [30]. All these previous results, along with our VL findings, reinforce the notion that diabetic individuals, even without DSP, have difficulty producing proper shock absorption at early stance [2,3,30], and they exhibit greater participation of the knee in this function. Interestingly, this pattern seems to be more pronounced in subjects with severe neuropathy, as they displayed an increase in VL relative magnitude, an indication of even greater participation of the knee joint in this gait cycle phase.

All the diabetic groups in this study showed a lower relative magnitude of TA, suggesting impaired function at the heel strike phase when this muscle plays an essential role in controlling forefoot contact with the ground and in attenuating the initial impact. This poor TA function at heel strike can be associated with the previously described delayed VL pattern at this specific gait phase, in both the D and SN groups. This gait phase is characterized by impact absorption both at the heel and at the forefoot, and with these alterations in both pivotal muscles, the shock absorption mechanics may be compromised. This change in pretibial muscles was formerly shown by a delay in the instant of maximum TA activity [2,22]. Although our results agree with previous findings that have not shown any temporal difference between healthy adults and neuropathic patients, with or without previous ulceration [3,6], the previous studies did not measure the magnitude of the EMG signals as we did, and the lower TA activity observed in the present study could have a biomechanical effect on the shock absorption mechanism.

Diabetic individuals, both with and without neuropathy, are known to have reduced gait velocity, cadence, and stride length [31,32], which could explain the anticipation of TA onset in late stance that we observed, as toe-off would occur earlier, resulting in a short stride length. Still, this motor pattern, associated with the higher relative magnitude at late stance observed in diabetics without neuropathy, may explain the reported lower extension moments [5,33,34], joint stiffness [35-37], and reduced plantar flexion at late stance [6,34], all observed in this population. In addition, in patients with severe neuropathy, GM presented diminished relative magnitude and delayed peak activity, which would also contribute to this impaired role of propulsion at late stance. This temporal GM delay was previously described, even with imposed gait velocities [6], and has occurred only in individuals with a history of plantar ulceration [3], an indication of a worse state of disease, which corresponds with the more severe diabetics in our study.

The fuzzy model was successful in separating the different levels of DSP, leading to a better definition of the stage when biomechanical alterations occur, as well as an understanding of how they develop as neuropathy worsens. It is clear now that different muscles presented activation impairments in distinct stages of the disease progression, with the states of absent (D group) and early (MiN group) stages of neuropathy causing changes in shock absorption mechanisms, while later stages (MoN and SN groups) are associated with the addition of impairments in the propulsion phase of gait.

Therefore, biomechanical alterations should be more carefully analyzed in gait studies, considering not only the presence of DSP, but also the influence of the diabetes control and its status. It is important to highlight that this fuzzy model still needs to be validated, comparing its results with a gold standard method, such as nerve conduction studies, but it already has a high correlation with the results reached by human experts [17].

Although we found significant differences in muscle activity patterns, we cannot discard the possible influence of gait velocity on our results, as all subjects were instructed to walk at a self-selected cadence, and gait velocity was not controlled during the experiment. However, diabetic patients, with or without neuropathy, are known to walk slowly, with a low cadence and small steps [29,38], and previous studies that controlled gait speed and cadence [6,8] found that faster walking caused an overall anticipation of muscle activity. Another limitation of this study was the higher body mass index found in the diabetic groups, a factor that could cause differences in EMG because of skin impedance and low-pass filtering effect. However, with their mean value falling under the same classification (overweight), the effect of resistance to electrical current would be the same throughout the groups. In addition, a higher body mass index is a common characteristic in these patients; therefore, recruiting this high number of patients with lower body mass index would be very difficult to manage.

The biomechanical alterations during diabetic patients’ gait still have many unanswered questions, but it is clear that neuropathy degree must be taken into account to analyze them, and the same applies in health systems’ treatment and prevention strategies for this population. In order to reach more decisive conclusions, future studies on muscle activity should capture a larger area of the muscle, as there is heterogeneity in EMG signals throughout the muscle tissue due to the disease [25,39]. In addition, other methods of signal processing should be used, possibly extracting information on conduction velocity and spectral properties.

## Conclusion

The activity levels of lower limb muscles during gait changed at distinct severity degrees of DSP. There is a delay in VL peak activity and reduced TA relative magnitude that are present even before neurological involvement, suggesting an impairment in the shock absorption mechanism at the ankle and a higher dependence on the knee´s absorptive function in the weight acceptance phase of gait in the early stages of the disease. With the onset of neuropathy, this proximal compensation continues, with an increase in VL relative magnitude. At late stance, TA onset is anticipated even in the absence of DSP, with an intensification of this pattern in severe neuropathy state, along with peak delay and lower activation of GM in the moderate and severe groups.

DSP severity degree must be taken into account when analyzing the biomechanics of locomotion of diabetic patients, and we recommend the use of a fuzzy system to assess the state of disease, not only for research purposes, but also in the healthcare system.

## Abbreviations

EMG: Electromyography; DSP: Diabetic sensorimotor polyneuropathy; C: Control group; D: Diabetic patients without neuropathy; MiN: Mild neuropathy group; MoN: Moderate neuropathy group; SN: Severe neuropathy group; VL: Vastus lateralis; TA: Tibialis anterior; GM: Gastrocnemius medialis.

## Competing interests

The authors declare that they have no competing interests.

## Authors´ contribution

RW, CDS and MKB contributed to the design of the study, the analysis of the data and the interpretation of the results. APP and NRSO supervised the development of the fuzzy model. CFA contributed to the analysis of the data and the interpretation of the results. ICNS conceived the study and its design, coordinated and helped to draft the manuscript. All the authors have revised the manuscript and have given their final approval for publication.
